# Advanced glycation end-products and its soluble receptor are not independent predictors of incident dysglycaemia or metabolic syndrome in women with polycystic ovary syndrome: a prospective observational study

**DOI:** 10.1186/s12958-023-01093-7

**Published:** 2023-05-10

**Authors:** Yu Wing Tong, Jennifer Ka Yee Ko, Karen Siu Ling Lam, Sidney Tam, Vivian Chi Yan Lee, Pak Chung Ho, Ernest Hung Yu Ng, Raymond Hang Wun Li

**Affiliations:** 1 Department of Obstetrics and Gynaecology, The University of Hong Kong, Queen Mary Hospital, Pokfulam, Hong Kong; 2 Department of Medicine, The University of Hong Kong, Queen Mary Hospital, Pokfulam, Hong Kong; 3 Department of Pathology, The University of Hong Kong, Queen Mary Hospital, Pokfulam, Hong Kong

**Keywords:** Advanced glycation end-products, Soluble receptor of advanced glycation end-products, Polycystic ovary syndrome, Dysglycaemia, Metabolic syndrome

## Abstract

**Background:**

To evaluate the association of serum advanced glycation end-products (AGEs) and its soluble receptor of AGE (sRAGE) levels with dysglycaemia and metabolic syndrome in women with polycystic ovary syndrome (PCOS).

**Methods:**

This was an analysis of a cohort of women with PCOS who were prospectively recruited for a longitudinal observational study on their endocrine and metabolic profile between January 2010 and December 2013. The association of serum AGEs and sRAGE levels with dysglycaemia and metabolic syndrome at the second-year visit (the index visit) and the sixth-year visit (the outcome visit) were determined. Comparisons of continuous variables between groups were made using the Mann–Whitney U-test. Spearman test was used for correlation analysis. Multivariate binary logistic regression analysis was employed to identify the factors independently associated with the outcome events.

**Results:**

A total of 329 women were analysed at the index visit. Significantly lower serum levels of sRAGE (both p < 0.001), but no significant difference in AGEs, were observed in those with dysglycaemia or metabolic syndrome. At the outcome visit, those with incident metabolic syndrome had a significantly lower initial serum sRAGE levels (p = 0.008). The association of serum sRAGE with dysglycaemia and metabolic syndrome at the index visit was no longer significant in multivariate logistic regression after controlling for body mass index, free androgen index and homeostatic model assessment for insulin resistance (HOMA-IR). sRAGE was also not significantly associated with incident metabolic syndrome at the outcome visit on multivariate logistic regression.

**Conclusions:**

Serum sRAGE levels are significantly lower in women with PCOS who have dysglycaemia or metabolic syndrome, and in those developing incident metabolic syndrome in four years. However, it does not have a significant independent association with these outcome measures after adjusting for body mass index, free androgen index and HOMA-IR.

## Background

Polycystic ovary syndrome (PCOS) is a common disease in reproductive-age women, with a prevalence of up to 6–12% [1]. It is diagnosed based on two out of three criteria, namely menstrual irregularity, hyperandrogenism and polycystic ovaries on pelvic ultrasonography [2]. Apart from these, it is also strongly associated with insulin resistance and metabolic syndrome [3]. Therefore, care of women with PCOS essentially includes prevention, early detection and management of their metabolic problems [4]. For instance, the oral glucose tolerance test (OGTT) has been recommended as the screening method for dysglycaemia in women with PCOS, regardless of body mass index (BMI) or family history of diabetes mellitus (DM) [5].

Advanced glycation end-products (AGEs) are products of protein or lipid molecules formed from exposure to sugars in the blood. AGEs bind to specific receptors of AGE (RAGE) on the surface of different cells, resulting in oxidative stress. An increased serum level of AGEs has been found in patients with various diseases including chronic renal disease [6], rheumatoid arthritis [7], malignancy [8] and ageing [9]. It is believed that AGEs accumulate in these organs and cause oxidative stress and inflammatory damage. It is also one of the proposed mechanisms to cause tissue injury in multiple organs in diabetic patients. Previously, it was thought that AGEs are only the consequence of DM causing diabetic complications in different organs [10]. Soluble receptor for AGEs (sRAGE) is a form of receptor that is secreted extracellularly into the blood circulation. It can counteract the adverse systemic effects of AGEs as it binds circulating AGEs and prevents them from binding to RAGE and triggering cellular oxidative injury [11]. AGEs and sRAGE have been therefore proposed as biomarkers for the prediction of diabetic complications like peripheral artery disease [12]. Lately, they are also hypothesised as being the “common soil” of the pathophysiology of PCOS, insulin resistance and metabolic syndrome [13]. A study in the United States has shown that a single measurement of serum sRAGE can reliably assess the average sRAGE level over several years, supporting their use in epidemiologic studies [14]. Apart from its predictive value, it might also potentially be a therapeutic target for protecting patients from AGEs-mediated cell injury. Novel therapeutic use of sRAGE in mice to delay the progression of amyotrophic lateral sclerosis has been reported [15].

In women with PCOS, higher levels of serum AGEs have been detected compared to healthy women [16]. It is suggested that AGEs can alter granulosa and theca cell function and cause dysfunction in steroidogenesis and follicular development [17]. It has been suggested that AGEs interfere with luteinizing hormone action in granulosa cells, leading to abnormal activation of the ERK1/2 pathway which might contribute to the anovulation in PCOS patients [18]. Even in women with PCOS who were normoglycaemic, serum AGEs levels were positively associated with testosterone level, free androgen index, insulin level, and waist-to-hip ratio [19]. Although the detailed molecular effects of AGEs in PCOS are yet to be fully understood, accumulating evidence suggests that it has an important role in the pathophysiology of PCOS and metabolic diseases. Knowing that the incidence of dysglycaemia and metabolic syndrome is higher in women with PCOS, prediction of those who would have an increased risk of developing dysglycaemia and metabolic syndrome would have a significant benefit in their care, as those at higher metabolic risk may warrant a closer monitoring and earlier intervention. There is a lack of consensus on the optimal method to predict dysglycaemia and metabolic syndrome in PCOS women despite several guidelines suggesting regular assessment of glycaemic status [20]. Currently, there are limited data on the predictive performance of AGEs and sRAGE on the incidence of dysglycaemia and metabolic syndrome in women with PCOS.

In this study, we aimed to evaluate the association of serum AGEs and sRAGE levels with existing dysglycaemia and metabolic syndrome, and with the development of incident dysglycaemia and metabolic syndrome over a follow-up period of four years in women with PCOS.

## Methods

This is a longitudinal observational study on a cohort of women with PCOS who were prospectively recruited and followed up on their endocrine and metabolic profiles.

### Subject recruitment

Our study included women of Chinese ethnicity who were diagnosed with PCOS at the Department of Obstetrics and Gynaecology, Queen Mary Hospital, a university-affiliated tertiary hospital in Hong Kong, and the Family Planning Association of Hong Kong, a community sexual and reproductive health centre, between January 2010 and December 2013 [21]. PCOS was defined by the Rotterdam criteria, which required the presence of any two out of the following three criteria: (i) oligomenorrhoea/amenorrhoea; (ii) clinical or biochemical hyperandrogenism and (iii) polycystic ovary morphology on pelvic scanning [2]. Clinical hyperandrogenism was defined by subjectively bothersome acne, seborrhoea or hirsutism, and/or a modified Ferrimen-Gallway score of > = 5. Biochemical hyperandrogenism was defined by a serum total testosterone concentration of 1.6 nmol/L or above, or a free androgen index (FAI) of 5.0 or above. FAI was calculated as 100 times the serum total testosterone level divided by the serum sex hormone-binding globulin (SHBG) level. Transvaginal or transrectal ultrasonography was performed for antral follicle count to confirm polycystic ovary morphology. The ultrasound examinations were performed with a 7–9 MHz probe on the Voluson V730 PRO ultrasound machine (GE Medical, Zipf, Austria). Ethics approval was obtained from the Institutional Review Board of the University of Hong Kong/Hospital Authority Hong Kong West Cluster. All women gave written informed consent. They attended the research clinic every two years for monitoring of their endocrine and metabolic profile as detailed below.

### Clinical and biochemical assessment of subjects

At each visit, the recruited women attended the clinic within the first 5 days of spontaneous or progestogen-induced menstruation. Blood pressure, height, weight, and waist circumference were measured. A blood sample after an overnight fast of at least 8 h was checked for total testosterone, SHBG, glucose, insulin and lipid profile. They then had a 75 g OGTT with blood taken for glucose at fasting and 2 h. Recruited women were followed up every two-yearly to monitor their clinical and biochemical parameters.

Blood glucose was analysed by Cobas 8000 modular analyser (Roche Diagnostics, Indianapolis, IN), testosterone by Vitros 3600 immunodiagnostic system (Ortho Clinical Diagnostics Inc, USA) and SHBG by Immulite 2000 immunoassay system (Siemens Healthcare Diagnostics Inc, Malvern, PA). The Hitachi 717 analyzer (Boehringer Mannheim, Germany) was used for cholesterol and triglyceride measurements by the cholesterol oxidase/glycerol kinase methods. The HDL-C level was measured using the cholesterol oxidase method, after precipitation of the apolipoprotein B containing lipoproteins. Calculation of LDL-C level was done by the Friedwald Eq. [22]. The homeostatic model assessment for insulin resistance (HOMA-IR) was calculated as fasting glucose levels in mmol/L multiplied by fasting insulin levels in mIU/L divided by 22.5 [23].

### Diagnosis of dysglycaemia

The diagnoses of diabetes, impaired glucose tolerance and impaired fasting glucose were made based on the American Diabetes Association criteria for plasma glucose levels [24]. Diabetes was diagnosed if the fasting blood glucose was > = 7.0mmol/L or 2-h post-load glucose was > = 11.1 mmol/L. Impaired fasting glucose was diagnosed if the fasting blood glucose was 5.6–6.9 mmol/L. Impaired glucose tolerance was diagnosed if the 2-h post-load glucose was 7.8–11.1 mmol/L. Dysglycaemia was diagnosed if the patient had either diabetes, impaired fasting glucose or impaired glucose tolerance.

### Diagnosis of metabolic syndrome

A diagnosis of the metabolic syndrome was made based on the Joint Interim Statement of the International Diabetes Federation Task Force of Epidemiology and Prevention, National Heart, Lung and Blood Institute American Heart Association, World Heart Federation, International Atherosclerosis Society and International Association for the Study of Obesity. Metabolic syndrome was diagnosed if at least three out of the following five criteria were met: (i) waist circumference > 80 cm (Chinese women); (ii) triglycerides > = 1.7 mmol/L; (iii) HDL-cholesterol < = 1.29 mmol/L; (iv) systolic blood pressure > 130 mmHg or diastolic blood pressure > 85 mmHg; and (v) fasting glucose > = 5.6 mmol/L or known history of type 2 diabetes mellitus [25].

### Measurement of serum AGEs and sRAGE

In this current study, the second-year visit was taken as the index visit, and archived serum samples collected at this index visit were retrieved and assayed for AGEs and sRAGE by enzyme-linked immunosorbent assay (Biomatik, Ontario, Canada; catalogue numbers EKC32508 and EKF57154) according to the manufacturer’s manual. The intra- and inter-assay coefficients of variation of both assays were < 8% and < 10% respectively.

### Primary outcomes measures

The sixth-year visit (i.e. four years after the index visit) was taken as the outcome visit. The primary outcome measures were the occurrence of dysglycaemia and metabolic syndrome at the index visit, as well as incident dysglycaemia and incident metabolic syndrome at the outcome visit.

## Statistics analysis

Statistical tests were performed by SPSS (version 26, IBM Corporation, Armonk, NY). As most of the continuous variables were not normally distributed, non-parametric statistical tests were employed. Comparisons of continuous variables between groups were made using the Mann–Whitney U-test. The Spearman test was used for correlation analysis. Multivariate binary logistic regression analysis was employed to identify the factors independently associated with the outcome events, and included those factors which showed significant between-group differences, taking into consideration biological relevance and possible presence of multi-collinearity in univariate analyses. A p-value of less than 0.05 was considered statistically significant.

As for the sample size, all of the originally recruited cohorts who attended the second-year and sixth-year follow-ups were included in this analysis. As the multivariate logistic regression included four variables, a minimum number of 216 and 165 cases could achieve adequate statistical power for analyzing dysglycaemia and metabolic syndrome based on their respective incidences at the index visit according to the Long’s rule. Hence, our sample size of 329 at the index visit was considered adequate.

## Results

Out of 471 women who were diagnosed to have PCOS and recruited into the original study, 329 attended follow-up visits at the second year (the index visit) and 232 at the sixth year (the outcome visit) and were available for analysis. Of those attending the index visit, 204 (62.0%) had all features of hyperandrogenism, oligo-amenorrhoea and polycystic ovary morphology, 5 (1.5%) had hyperandrogenism and oligo-amenorrhoea, 1 (0.3%) had hyperandrogenism and polycystic ovary morphology, and 119 (36.2%) had oligo-amenorrhoea and polycystic ovary morphology only (shown in Table 1). Among the 329 patients analyzed at the index visit, 61 (18.5%) of them were diagnosed with dysglycaemia and 80 (24.3%) of them were diagnosed with metabolic syndrome. At the outcome visit, 19 (8.2%) patients were diagnosed with incident dysglycaemia (being normoglycaemic at the index visit) and 21 (9.1%) patients were diagnosed with incident metabolic syndrome (without metabolic syndrome at the index visit).

**Table 1:** Phenotype of women with PCOS included in this study

	Number (n = 329)	%
HA + OA + PCOM	204	62.0
HA + OA	5	1.5
HA + PCOM	1	0.3
OA + PCOM	119	36.2

HA: Hyperandrogenism (raised total testosterone, androstenedione or free androgen index, or hirsutism)

OA: Oligo/amenorrhoea (Cycle length > 35 days)

PCOM: Polycystic ovarian morphology (antral follicle count > = 12 or ovarian volume > 10 ml)

The correlation of serum AGEs and sRAGE with clinical-demographic parameters for the study participants are shown in Table 2. Serum AGEs showed a significant positive correlation with fasting glucose, total cholesterol, low-density lipoprotein-cholesterol (LDL-C) and triglycerides, and an inverse correlation with high-density lipoprotein-cholesterol (HDL-C). Serum sRAGE showed a significant positive correlation with SHBG and HDL-C, and inverse correlation with BMI, waist circumference, FAI, fasting glucose, 2-hour glucose, systolic and diastolic blood pressure, triglycerides and HOMA-IR.

**Table 2:** Correlation of serum AGEs and sRAGE with clinical-demographic parameters for women with PCOS

Parameter	AGEs		sRAGE	
	Spearman’s r	P value	Spearman’s r	P value
Age of women (years)	0.019	0.729	-0.79	0.152
Body mass index (kg/m^2^)	0.100	0.072	-0.435	< 0.001*
Waist circumference (cm)	0.089	0.108	-0.459	< 0.001*
Antral follicle count	0.082	0.143	0.051	0.360
Testosterone (nmol/L)	-0.055	0.320	0.093	0.093
SHBG (nmol/L)	-0.074	0.181	0.355	< 0.001*
Free androgen index	0.021	0.706	-0.194	< 0.001*
Fasting glucose (mmol/L)	0.109	0.048*	-0.144	0.009*
2-Hour glucose (mmol/L)	0.041	0.464	-0.234	< 0.001*
HOMA-IR	-0.010	0.886	-0.290	< 0.001*
Systolic blood pressure (mmHg)	0.003	0.962	-0.249	< 0.001*
Diastolic blood pressure (mmHg)	-0.044	0.427	-0.180	0.001*
Total cholesterol (mmol/L)	0.139	0.011*	-0.026	0.640
HDL-C (mmol/L)	-0.136	0.013*	0.310	< 0.001*
LDL-C (mmol/L)	0.183	0.001*	-0.076	0.171
Triglycerides (mmol/L)	0.111	0.044*	-0.238	< 0.001*

*Statistically significant (p < 0.05)

The demographic and clinical characteristics of the subjects at the index visit are shown in Table 3. These were compared between those with and without dysglycaemia and metabolic syndrome respectively. At the index visit, significantly lower serum levels of sRAGE (p < 0.001), and higher BMI (p < 0.001), FAI (p < 0.001) and HOMA-IR (p < 0.001) were observed both in those with dysglycaemia and those with metabolic syndrome compared with those without. There was no significant difference in serum AGEs between those with or without dysglycaemia (p = 0.260) or metabolic syndrome (p = 0.190). Multivariate logistic regression analyses for factors associated with dysglycaemia and metabolic syndrome at the index visit are shown in Table 4. Serum sRAGE was not significantly associated with both dysglycaemia and metabolic syndrome at the index visit after controlling for BMI, FAI and HOMA-IR.

**Table 3:** Demographic and clinical characteristics at the index visit. Continuous data are expressed as median (25th – 75th percentile), while categorical data are expressed as N(%)

Parameter	Overall	Dysglycaemia at index visit			Metabolic syndrome at index visit		
		No (n = 268)	Yes (n = 61)	P value	No (n = 249)	Yes (n = 80)	P value
Age (years)	32 (28.5–35.5)	32 (29–35)	33 (28–36)	0.306	32 (29–35)	33 (28–36)	0.398
BMI (kg/m^2^)	22.8 (20.7–26.8)	22.5 (20.3–25.4)	28.3 (24.0–30.8)	< 0.001*	22.1 (20.0–24.2)	29.3 (26.9–34.0)	< 0.001*
Waist circumference (cm)	77.3 (71.0–85.8)	76 (71–83)	89 (78–94)	< 0.001*	75 (70–80)	91 (86–99)	< 0.001*
Total testosterone (nmol/L)	1.2 (0.9–1.6)	1.2 (0.9–1.6)	1.4 (1.0–1.9)	0.038*	1.2 (0.8–1.6)	1.4 (0.9–1.8)	0.022*
SHBG	36 (22–52)	40 (27–55)	21 (15–32)	< 0.001*	42 (30- − 58)	19 (13–28)	< 0.001*
Free androgen index	3.3 (1.8–6.0)	3.0 (1.7–5.5)	5.9 (3.2–14.2)	< 0.001*	2.6 (1.6–4.7)	7.0 (4.3–12.9)	< 0.001*
Fasting glucose (nmol/L)	4.7 (4.4–4.9)	4.6 (4.4–4.9)	5.2 (4.6–5.8)	< 0.001*	4.6 (4.4. – 4.8)	5.0 (4.6–5.5)	< 0.001*
2-Hour glucose (nmol/L)	5.1 (4.5–6.4)	4.8 (4.4–5.6)	7.8 (6.6–9.6)	< 0.001*	4.9 (4.3–5.6)	6.6 (5.1–8.3)	< 0.001*
HOMA-IR	1.5 (1.0–2.4)	1.3 (0.9–1.9)	3.1 (1.5–5.6)	< 0.001*	1.2 (0.9–1.8)	3.3 (2.0–5.3)	< 0.001*
Systolic blood pressure (mm/Hg)	114 (105–127)	111 (104–122)	126 (113–134)	< 0.001*	110 (103–121)	129 (116–138)	< 0.001*
Diastolic blood pressure (mm/Hg)	67 (61–75)	66 (60–73)	76 (65–80)	< 0.001*	65 (59–71)	76 (69–83)	< 0.001*
Total cholesterol (mmol/L)	4.5 (4.0-5.1)	4.5 (4.0–5.1)	4.6 (4.2–5.2)	0.063	4.5 (4.0–5.0)	4.9 (4.2–5.3)	0.002*
HDL cholesterol (mmol/L)	1.4 (1.2–1.7)	1.5 (1.3–1.7)	1.2 (1.0–1.5)	< 0.001*	1.5 (1.3–1.8)	1.1 (1.0–1.2)	< 0.001*
LDL-cholesterol (mmol/L)	2.6 (2.2–3.0)	2.6 (2.1–3.0)	2.7 (2.4–3.3)	0.020*	2.6 (2.1–2.9)	2.9 (2.6–3.5)	< 0.001*
Triglycerides (mmol/L)	0.9 (0.7–1.2)	0.8 (0.6–1.1)	1.2 (0.9–1.7)	< 0.001*	0.8 (0.6–1.0)	1.5 (1.0–2.1)	< 0.001*
AGEs (µg/ml)	14.8 (10.7–19.5)	14.6 (10.3–19.4)	15.3 (11.2–21.3)	0.260	14.6 (10.3–19.2)	15.8 (11.5–20.7)	0.190
sRAGE (pg/ml)	595.7 (471.3–796.3)	615.7 (492.5–821.0)	493.4 (401.2–658.6)	< 0.001*	650.3 (532.1–834.9)	463.7 (393.0–560.6)	< 0.001*

*Statistically significant (p < 0.05)

**Table 4:** Multivariate binary logistic regression analyses for factors associated with dysglycaemia and metabolic syndrome at the index visit.

Factor	Odds ratio (95% confidence interval)	P value
**(a) Dysglycaemia**:		
**Serum sRAGE level (pg/ml)**	0.999 (0.997–1.000)	0.125
**Body mass index (kg/m** ^**2**^ **)**	1.060 (0.992–1.132)	0.083
**Free androgen index**	1.071 (1.011–1.134)	0.020*
**HOMA-IR**	1.220 (1.066–1.396)	0.004*
**(b) Metabolic syndrome**:		
**Serum sRAGE level (pg/ml)**	0.999 (0.997–1.001)	0.191
**Body mass index (kg/m** ^**2**^ **)**	1.383 (1.243–1.539)	< 0.001*
**Free androgen index**	1.039 (0.967–1.117)	0.297
**HOMA-IR**	1.251 (1.050–1.489)	0.012*

*Statistically significant (p < 0.05)

At the outcome visit, those with incident metabolic syndrome had a significantly lower serum sRAGE level (p = 0.008), as well as higher BMI (p < 0.001) and HOMA-IR (p = 0.005) at the index visit than those without (shown in Table 5). Only BMI (p < 0.001), but not serum sRAGE (p = 0.536) nor HOMA-IR (p = 0.900), was significantly associated with incident metabolic syndrome in multivariate binary logistic regression analysis (shown in Table 6).

**Table 5:** Comparison of clinical characteristics at the index visit between those with and without incident dysglycaemia and incident metabolic syndrome at the outcome visit

Parameter	Incident dysglycaemia^			Incident metabolic syndrome^		
	No (n = 172)	Yes (n = 19)	P value	No (n = 160)	Yes (n = 21)	P value
BMI (kg/m^2^)	22.4 (20.5–25.0)	25.7 (20.8–31.1)	0.005*	22.0 (20.1–23.4)	25.7 (22.7–28.8)	< 0.001*
Free androgen index	3.0 (1.8–5.0)	5.3 (1.8–6.5)	0.102	2.7 (1.7–4.3)	3.6 (1.7–10.5)	0.200
HOMA-IR	1.3 (0.9–1.9)	1.6 (1.1–2.1)	0.027*	1.2 (0.9–1.7)	1.6 (1.4–2.1)	0.005*
AGEs (µg/ml)	14.5 (10.2–18.7)	17.4 (11.8–21.2)	0.110	14.7 (10.2–19.4)	14.0 (10.6–16.1)	0.481
sRAGE (pg/ml)	629.2 (492.5–821.0)	581.1 (436.9–866.0)	0.656	661.2 (553.5–836.9)	494.1 (469.2–700.3)	0.008*

^Those with existing dysglycaemia (n = 41) or metabolic syndrome (n = 51) at the index visit were excluded from the respective analyses

*Statistically significant (p < 0.05)

**Table 6:** Multivariate binary logistic regression analysis for factors predicting incident metabolic syndrome at the outcome visit. Those with existing metabolic syndrome at the index visit were excluded from analysis

Factor	Odds ratio (95% confidence interval)	P value
**Serum sRAGE level**	0.999 (0.997–1.002)	0.536
**Body mass index**	1.319 (1.132–1.535)	< 0.001*
**HOMA-IR**	0.976 (0.664–1.434)	0.900

*Statistically significant (p < 0.05)

## Discussion

PCOS is highly associated with dysglycaemia and metabolic syndrome. Among our cohort of women with PCOS, the prevalence of dysglycaemia at the index visit was 18.5% and that of metabolic syndrome was 24.3%. A meta-analysis published in 2018 including 4530 studies on PCOS women found that they have an increased risk of metabolic syndrome (odds ratio 3.35) [26]. DM and metabolic syndrome are important modifiable risk factors for all-cause mortality [27, 28]. Early detection and treatment can reduce both morbidity and mortality [29]. AGEs and sRAGE are increasingly recognized as important molecules mediating adverse complications of these diseases [30]. Studies have suggested that diet modification including choice and preparation of food might help to reduce AGEs levels and possibly improve control of AGEs-implicated diseases [31]. For example, frying, broiling, grilling, and roasting resulted in more dietary AGEs compared to boiling, poaching, stewing, and steaming. And grains, legumes, bread, vegetables, fruits, and milk are the food of choice with the lowest dietary AGEs. It was also found that angiotensin-converting enzyme inhibition might help to reduce the serum level of AGEs [32]. Future development should focus on their diagnostic and therapeutic applications.

In our current study, sRAGE showed a significant association with BMI, waist circumference, SHBG, FAI, fasting glucose, 2-hour glucose, systolic blood pressure, HDL-C and triglycerides and HOMA-IR at the index visit. A similar inverse correlation between sRAGE and BMI, waist circumference and triglycerides was reported earlier in a Taiwanese study on male and female adolescents [33]. Another Shanghai study on women with PCOS also showed a similar inverse correlation between sRAGE and BMI, FAI and waist circumference [34]. To our knowledge, ours is the first to investigate the association of AGEs and sRAGE with dysglycaemia and metabolic syndrome in women with PCOS, which revealed that serum AGEs has little predictive role in this regard, while serum sRAGE level was not an independent predictor of prevalent dysglycaemia or metabolic syndrome after controlling for BMI, FAI and HOMA-IR, nor of incident dysglycaemia or metabolic syndrome upon longitudinal follow-up after controlling for BMI and HOMA-IR. The association of sRAGE with prevalent dysglycaemia and metabolic syndrome could merely reflect the association between sRAGE level and insulin resistance, obesity and/or hyperandrogenism. On the other hand, the prospective inverse association of sRAGE with incident metabolic syndrome could largely be explained by its inverse relationship with obesity.

The main limitation of this study was its relatively short follow-up period of four years. We used the second visit of our cohort as the index visit, as this subsidiary study on advanced glycation end-products was not part of the original primary study, and some of the serum samples from the baseline recruitment visit were no longer available when this subsidiary study was planned, while a complete biobank of those attending the second-year follow-up was available. Dysglycaemia and metabolic syndrome are chronic diseases that might develop over a longer term. A longer prospective follow-up might be worthwhile to give more data to support the findings. Moreover, the AGEs and sRAGE levels can be affected by various lifestyle factors such as diet, cigarette smoking and exercise. This information was not recorded in detail in our study, which was another limitation. These all could be potential confounding factors. As these lifestyle factors could have been modified throughout the follow-up period, a single measurement at the index visit may not reflect the average AGEs and sRAGE level throughout the follow-up period. This may explain the limitation of using these markers for predicting incident metabolic outcomes in longitudinal follow-up. Furthermore, our recruitment included Chinese women only. The Asian population, in general, have a lower BMI than many other populations [35–38]. On the other hand, the Western diet is known to have a higher proportion of processed food and sugar, which might contribute to a higher intake of exogenous AGEs [39]. Therefore, this might significantly limit the generalizability to other ethnic groups.

## Conclusions

Serum sRAGE levels are significantly lower in women with PCOS who have dysglycaemia or metabolic syndrome, and in those developing incident metabolic syndrome over a four-year follow-up. However, it does not have a significant independent association with these outcome measures after adjusting for BMI, FAI and HOMA-IR. A larger-scale international prospective study with a longer follow-up might be helpful in providing more data to verify the findings.

## Data Availability

The datasets analysed during the current study are available from the corresponding author on reasonable request.

## Abbreviations

AGEs: Serum advanced glycation end-products
sRAGE: Soluble receptor of AGE
PCOS: Polycystic ovary syndrome
HOMA-IR: Homeostatic model assessment for insulin resistance
OGTT: Oral glucose tolerance test
BMI: Body mass index
DM: Diabetes mellitus
RAGE: Receptors of AGE
FAI: Free androgen index
SHBG: Serum sex hormone-binding globulin
HDL-C: High density lipoprotein-cholesterol
LDL-C: Low density lipoprotein-cholesterol
